# Transcriptional Activation of Inflammatory Genes: Mechanistic Insight into Selectivity and Diversity

**DOI:** 10.3390/biom5043087

**Published:** 2015-11-11

**Authors:** Afsar U. Ahmed, Bryan R. G. Williams, Gregory E. Hannigan

**Affiliations:** The Centre for Cancer Research, Hudson Institute of Medical Research, 27–31 Wright Street, Clayton VIC 3168, Australia; E-Mail: afsar.ahmed@hudson.org.au

**Keywords:** inflammation, pro-inflammatory genes, transcriptional activation, NF-κB, MAPK, ubiquitination, chromatin structures, nucleosome remodeling, epigenetic markers

## Abstract

Acute inflammation, an integral part of host defence and immunity, is a highly conserved cellular response to pathogens and other harmful stimuli. An inflammatory stimulation triggers transcriptional activation of selective pro-inflammatory genes that carry out specific functions such as anti-microbial activity or tissue healing. Based on the nature of inflammatory stimuli, an extensive exploitation of selective transcriptional activations of pro-inflammatory genes is performed by the host to ensure a defined inflammatory response. Inflammatory signal transductions are initiated by the recognition of inflammatory stimuli by transmembrane receptors, followed by the transmission of the signals to the nucleus for differential gene activations. The differential transcriptional activation of pro-inflammatory genes is precisely controlled by the selective binding of transcription factors to the promoters of these genes. Among a number of transcription factors identified to date, NF-κB still remains the most prominent and studied factor for its diverse range of selective transcriptional activities. Differential transcriptional activities of NF-κB are dictated by post-translational modifications, specificities in dimer formation, and variability in activation kinetics. Apart from the differential functions of transcription factors, the transcriptional activation of selective pro-inflammatory genes is also governed by chromatin structures, epigenetic markers, and other regulators as the field is continuously expanding.

## 1. Introduction

The survival of all living organisms relies on their ability to respond to a wide range of harmful stimuli such as microbial infection, environmental stresses, and tissue injures. The responses are initiated by immune mechanisms designed to detect and eliminate foreign pathogens, as well as heal damaged tissues. The effective responses by the immune system are mediated by the activation of signal transduction pathways leading to the altered expression of a subset of genes that are required to combat harmful consequences. Therefore, the expression patterns of selective genes at the transcriptional level are of central importance for the host defence mechanism.

Inflammation is one of the highly conserved and beneficial responses evolved in higher organisms in response to pathogens and other harmful stimuli. When a host with a functional innate immune system encounters foreign pathogens or tissue injuries, the inflammatory response initiates within minutes. Acute inflammation is therefore considered as the first line of host defence and an integral part of innate immunity. The inflammatory response triggers transcriptional activation of numerous genes, which carry out diverse physiological functions ranging from initiation of antimicrobial activities to the development of acquired immunity. Some of these gene products, such as antimicrobial peptides, directly target pathogenic microorganisms, while others, such as inflammatory cytokines and chemokines, activate the host defence by recruiting immune cells to the site of infection, as well as initiating healing of the injured tissues. Due to the enormous variety of pathogenic microorganisms and harmful stimuli routinely experienced by an organism, the inflammatory response involves an extensive exploitation of the transcriptional activation of selective genes intended for specific antimicrobial defence and tissue repairs. For example, bacterial pathogens induce the expression of inflammatory cytokines including tumour necrosis factor alpha (TNF-α), interleukin-1 (IL-1) and interleukin-6 (IL-6), as well as chemokines such as chemokine (C-C motif) ligand 2 (CCL2, also known as monocyte chemotactic protein 1, MCP-1), and chemokine (C-X-C motif) ligand 8 (CXCL8, also known as IL-8). On the other hand, viral infections induce type-1 interferons (IFN-α, IFN-β, IFN-γ), and parasitic worm infections or exposure to allergens are associated with the expression of histamine, IL-4, IL-5 and IL-13 [1].

This review focuses on the diversity of molecular mechanisms underlying the selective transcription of inflammatory genes. Apart from differential transcriptional activation, stimulus-dependent inflammatory signals also active the inflammasome, a molecular complex of several proteins that cleaves pro-IL-1β and pro-IL-18 into active IL-1β and IL-18. Since the latter has been reviewed in detail elsewhere [2], it will not be discussed further in this article.

## 2. Inflammation and Cellular Homeostasis

The main purpose of inflammation is to protect the host from any damaging consequences to cellular homeostasis. Once the inflammatory stimuli and sequelae have subsided, the acute inflammatory response is terminated. The resolution of inflammation, which includes the termination of inflammatory responses and the restoration of the homeostatic state, is in fact an integral part of the inflammatory process and needs to be carefully controlled. As an adaptive response to harmful stimuli, the inflammatory response is not only associated with host side effects, but is also maintained at the expense of normal physiological activities. Therefore, the duration of an inflammatory response is precisely regulated in order to avoid any unfavorable consequences to the host. Down-regulation of the response occurs via elaboration of anti-inflammatory molecules, such as IL-10, transforming growth factor-β (TGF-β), and glucocorticoids, which act to minimize the risk of excessive inflammation [1]. If the inflammatory condition persists without any resolution, a chronic inflammatory state develops. Chronic inflammation is generally triggered by chronic infection, unrepaired tissue damage, or persistent allergens [3,4]. However, for the majority of chronic inflammatory diseases (such as rheumatoid arthritis, type 2 diabetes, asthma, atherosclerosis, and cancer) the molecular triggers have yet to be defined, thus molecular mechanisms underlying these diseases remain largely unresolved. Based on the link between chronic inflammation and disease, the strategies for anti-inflammatory drug development hold much promise for therapeutic interventions. Accordingly, since excess levels of TNF-α are a distinctive feature of many inflammatory disorders, several anti-TNF-α antagonists have been developed and shown to be effective in a variety of autoimmune inflammatory diseases, including rheumatoid arthritis, ankylosing spondylitis, psoriatic arthritis, plaque psoriasis, juvenile idiopathic arthritis, ulcerative colitis, and Crohn’s disease [5,6].

## 3. Inflammatory Signal Transductions

Inflammatory signals are initiated by the recognition of inflammatory stimuli by specific transmembrane and intracellular receptors, called pattern-recognition receptors (PRRs). PRRs are germ-line encoded receptors that can specifically sense either pathogenic microorganisms or any cellular damage, and are expressed by cells of both the innate and adaptive immune systems [7]. Pathogen recognition by PRRs is based on conserved molecular structures called pathogen-associated molecular patterns (PAMPs), or endogenous molecules derived from tissue injuries, called damage-associated molecular patterns (DAMPs). There are several categories of PRRs, defined by their specificities for PAMPS and DAMPS. For example, Toll-like receptors (TLRs) recognize a wide variety of PAMPs from bacteria, fungi, parasites and viruses; C-type lectin receptors (CLRs) bind to carbohydrates in calcium-dependent manner; RIG-I-like receptors (RLRs) are intracellular receptors involved in the recognition of viruses; and NOD-like receptors (NLRs) are intracellular receptors that recognize cytoplasmic PAMPs and/or DAMPs.

Upon interaction with their cognate ligands, PRRs transduce signals to the cell nucleus to effect differential activation of gene transcription. Selectivity in the transcriptional regulation of these genes is largely dictated by the differences in the signal transduction pathways, which are exclusively governed by the nature of the stimuli initiating the PRR activation. In response to an inflammatory stimulus, activated genes have been divided into two categories based on the pattern of their transcriptional activation: primary and secondary response genes. Primary response genes, also referred to as immediate early genes, are activated rapidly after stimulation, whereas activation of secondary response genes is much slower [8,9,10]. It is the requirement for de novo protein synthesis that distinguishes secondary response genes from the immediate early genes, as the latter do not depend on new protein synthesis for activation.

**Figure 1:**
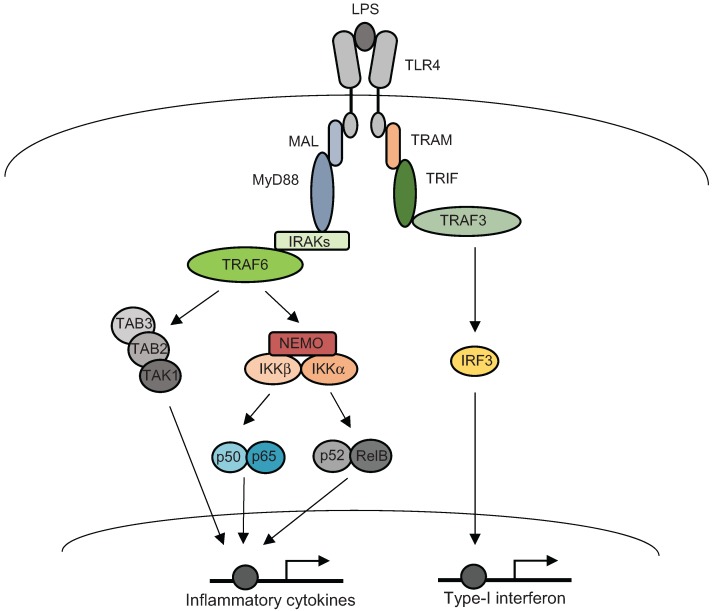
Toll-like receptors (TLR) 4-mediated signaling of innate immune responses. Binding of lipopolysaccharide (LPS) to TLR4 initiates a signaling complex by the recruitment of MyD88 and TIR domain-containing adaptor protein inducing IFN-β (TRIF), to the receptor. The MyD88-dependent signaling pathway activates transcriptional activation of pro-inflammatory cytokines via the activation of both NF-κB and mitogen-activated protein kinase (MAPK) s. The MyD88-independent pathway is mediated by TRIF and culminates in the production of type-1 interferons (IFN).

The discovery of the TLRs provided significant insight into how the host initiates inflammatory responses against microbial pathogens [11]. TLRs are type one transmembrane proteins characterized by an intracellular Toll-IL-1 receptor (TIR) domain and extracellular leucine-rich repeats (LRR) motif that recognize specific ligands. Being the prime receptor for bacterially derived lipopolysaccharide (LPS), TLR4 is the most studied among TLRs. Once activated, TLR4 homodimerizes and initiates the downstream signalling cascades by recruiting TIR-containing cytoplasmic adaptor proteins through a TIR-TIR interaction. Most TLRs signal via the universal adaptor protein, myeloid differentiation primary-response protein 88 (MyD88), with the exception of TLR3, which uses TIR domain-containing adaptor protein inducing IFN-β (TRIF), and TLR4, which signals via both MyD88 and TRIF. The signaling pathways emerging from TLR4 can be divided into two categories: MyD88-dependent and MyD88-independent pathways [12]. The MyD88-dependent TLR4 pathway mediates the early response to LPS. This pathway is facilitated by MyD88-adaptor like (Mal), a bridging adaptor required for TLR4-MyD88 interaction. Once bound to the complex, the death domain (DD) of MyD88 recruits IL-1 receptor associated kinases (IRAK1, IRAK2 and IRAK4) via DD-DD interactions. IRAK4 then interacts with IRAK1 to induce its phosphorylation. Phosphorylated IRAK4 dissociates from the complex and interacts with TNF receptor-associated factor six (TRAF6). The interaction of IRAK4 with TRAF6 facilitates the early induction of inflammatory gene transcription via the activation of transcription factors. On the other hand, the MyD88-independent pathway constitutes the late response to LPS and results in the expression of type-1 IFN. MyD88-independent pathway is mediated by TIR domain-containing adaptor inducing IFN-β (TRIF) and the TLR4-TRIF interaction is mediated by a bridging adaptor, TRIF-related adaptor molecule (TRAM). The N-terminus of TRIF recruits TRAF-3 and phosphorylates IRF3, leading to the induction of type-1 IFN [12,13] (Figure 1).

The differential regulation of gene activation at the transcriptional level is indicative of a precisely controlled binding of selective transcription factors to DNA motifs at the promoter of these genes. In fact, the efforts to identify transcription factors and their corresponding DNA binding motifs on target genes began decades ago. The nuclear factor κ(kappa)-light-chain-enhancer of activated B cells (NF-κB) was the first transcription factor identified whose binding to DNA is induced by a post-translational mechanism [14]. NF-κB is a robustly activated transcription factor, responsive to a number of inflammatory extracellular stimuli and its activity is regulated via post-translational mechanisms independent of new protein synthesis. NF-κB is one of the most well studied transcription factors and a great deal of knowledge of eukaryotic transcriptional mechanisms has been revealed through studies on NF-κB. The mammalian NF-κB family consists of five members: p65/RelA, RelB, c-Rel, p50 and p52, which share structural homology in their N termini with the retroviral oncoprotein v-Rel. via this rel homology region (RHR) they form stable homo- and/or heterodimers. NF-κB signaling is tightly controlled by a family of ankyrin-repeat containing inhibitory proteins (IκBs—IκBα, IκBβ and IκBε) or the precursors for p50 and p52, called p105 and p100, respectively, which also contain an IκB-like ankyrin repeat domain at their C-terminus. In resting cells, NF-κB is inactive and retained in the cytoplasm due to its association with IκB proteins. Following stimulation, NF-κB dimers are detached from IκB proteins and translocated to the nucleus to activate gene expression. The dissociation of NF-κB from IκB proteins is mediated either by phosphorylation of IκBs followed by ubiquitination and proteasome-mediated degradation, or by induced proteolytic cleavage of the ankyrin-repeat domain of p105 and p100. The signaling cascades leading to dissociation of NF-κB from IκB proteins is mediated by the IκB kinase complex, which consists of two catalytic subunits, IKKα and IKKβ. Among the NF-κB members, only p65/RelA, RelB and c-Rel contain transcriptional activation domains at their C-terminal [10,15,16,17,18,19]. The activation of IKKβ leads to the canonical NF-κB pathway, by initiating phosphorylation of IκBα and thereby facilitating nuclear translocation of the p50-RelA complex. Unlike the canonical pathway, which depends on the degradation of IκB for the nuclear translocation of the p50/RelA (p65) complex, the non-canonical NF-κB pathway is initiated by the activation of the p52/RelB complex and is dependent on the inducible processing of NF-κB2 precursor protein, p100. The processing of p100 is facilitated by NF-κB-inducing kinase (NIK) through IKKα activation. Therefore, the central component of the non-canonical pathway is NIK, which is otherwise subjected to continuous degradation under unstimulated conditions [20] (Figure 1).

In conjunction with the activation of NF-κB, the stimulation of TLRs also activates major mitogen-activated protein kinase (MAPK) subfamilies: the extracellular signal-regulated kinase (ERK), p38 and Jun N-terminal kinase (JNK) [21]. Following an inflammatory stimulation, MAPK activation induces the expression of multiple genes that together regulate the inflammatory response. Different MAPKs have distinct roles in transmitting the receptor-proximal signals to the transcriptional activation of selective genes via the phosphorylation of a range of down-stream substrates. The key step to the initiation of MAPK signaling is the activation of the essential MAPK kinase kinase (MAP3K). For example, the TGF-β-activated kinase 1 (TAK1) is a prominent MAP3K, which is critical for the activation of p38 and JNK following TLR or TNF stimulation. TAK1-binding protein 1 (TAB1), TAB2 and TAB3 play integrated roles in many TAK1-mediated functions (Figure 1). The other well-recognized MAP3Ks are the apoptosis signal-regulating kinase 1 (ASK1) and MAPK/ERK kinase kinase 3 (MEKK3) [21].

Following the discovery of NF-κB, a number of other transcription factors were identified that can also be activated by inflammatory stimuli via post-translational mechanisms, such as phosphorylation or dephosphorylation of these factors or their inhibitors. For example, activator protein-1 (AP-1), cyclic-AMP (cAMP) response element binding protein (CREB), serum responses factor (SRF), and the associated ternary complex factors (TCFs) [10,19]. Most of the transcription factors activated post-translationally contribute to the expression of the primary response genes. On the other hand, several other transcription factors were induced at the transcriptional level by inflammatory stimuli and these transcriptionally activated factors are mainly required for the expression of secondary response genes [10]. In certain cases, inflammatory stimuli activate transcriptional repressive pathways. An example of this is the post-translational modification of promyelocytic leukaemia zinc finger protein (PLZF) in response to TLR or TNF-α signaling, which results in calcium/calmodulin-dependent protein kinase (CaMK2) activation of the type-B histone acetylase-1 (HAT1) and acetylation of PLZF. This then promotes the assembly of a repressor complex consisting of histone deacetylase 3 (HDAC3) and NF-κB p50, thereby limiting NF-κB dependent transcriptional response [22,23]. Epigenetic control of chromatin states, such as that invoked by TLR signaling of PLZF, is discussed later in this review.

Even though a transcription factor binds to the promoter of a defined set of genes through a specific DNA motif, the selective activation of most genes is dependent on the synergistic effect of multiple transcription factors. Many inflammatory genes contain more than one DNA binding motif recognized by multiple transcriptions factors, and the binding of each and every transcription factor to its corresponding DNA motif is indispensable for the activation of these genes [24]. For example, the transcriptional activation of human IFN-β by Sendai virus is mediated by the assembly of a multi-protein complex, called an enhanceosome, formed by inducible transcription factors including key factors such as NF-κB, IRF3/IRF7 and ATF2 [25]. The activation of IFN-β gene mediated by cooperative binding of multiple DNA-binding proteins and each base pair with its 55 bp promoter region is involved in DNA-protein binding. Interestingly, such cooperative regulations of gene activation are not maintained by direct protein-protein interactions between these transcription factors, as these factors are rarely seen to be bound simultaneously to their specific DNA motifs. In fact, during cooperative activation of genes, the binding of these transcription factors to their corresponding DNA motifs within a single promoter proceeds transiently in a sequential and dynamic manner [26,27,28]. It is the conformational changes in the DNA structure that largely govern the sequential binding of these transcription factors. The binding of one transcription factor to its corresponding DNA motif induces a conformational change in DNA structure and thereby subsequently facilitates the binding of another factor to the adjacent or even the overlapping site on the same DNA motif [29,30].

## 4. Post-Translational Modifications of NF-κB

Despite the discovery of many transcription factors involved in the selective regulation of pro-inflammatory gene activations, NF-κB still remains at the forefront of eukaryotic transcriptional mechanisms because of its predominant role in inducing gene expression in response to a wide variety of stimuli in all cell types. Due to the extensive range of contributions of NF-κB, selective regulatory mechanisms are in place to ensure the activation of only a subset of genes in response to a defined stimulus. Although the NF-κB activation is initiated by its inducible nuclear translocation and binding to the corresponding DNA motifs, a number of different regulatory layers are involved for differential activation of target genes.

**Figure 2:**
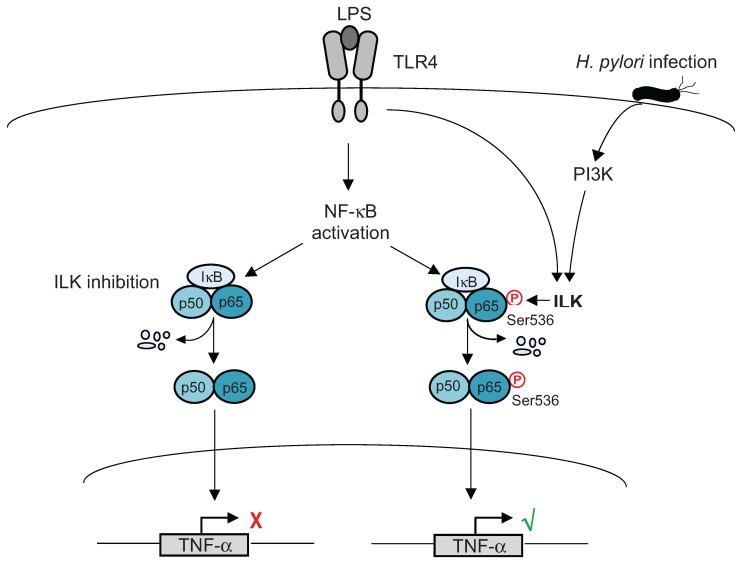
Integrin-linked kinase (ILK) modulates p65 Ser536 phosphorylation and tumour necrosis factor alpha (TNF-α) production in response to LPS and *H. pylori* infection. For *H. pylori* infection, the ILK-mediated role is PI3K dependent. Inhibition of ILK (via genetic knockdown or small molecule inhibitor treatment) prevents LPS or *H. pylori*-induced p65 Ser536 phosphorylation, but IκB degradation and nuclear translocation remain unaffected [42].

Post-translational modifications such as phosphorylation and acetylation of NF-κB proteins comprise a critical step towards the induction of many genes and the type of these modifications is dependent on specific stimuli [31,32]. One of the earliest descriptions of post-translational modifications of NF-κB proteins came from RelA (p65), which is also one of the most studied NF-κB proteins in terms of its post-translational modifications critical for selective gene activation. Initial studies showed that, during IκBα degradation, p65 is phosphorylated at Ser276 by protein kinase A (PKA) and this phosphorylation facilitates active transcription by mediating the recruitment of CREB-binding protein (CBP)/p300 to p65 [33,34]. A selective role for RelA Ser276 phosphorylation was demonstrated by the observation that targeted mutation of RelA Ser276 impairs the activation of some but not all NF-κB target genes [35]. Earlier studies also demonstrated that IKKβ plays an essential role in RelA phosphorylation at Ser536 in response to either TNF-α or LPS stimulation [36,37]. Several studies in recent years have shown that the phosphorylation of p65 at Ser536 by IKKβ augments the transactivation potential of p65 through a distinct, IκBα-independent mechanism [37,38,39,40,41]. In this pathway, the nuclear translocation of phospho(Ser536)-p65 is not dependent on IκBα degradation, but is critical for the regulation of a distinct set of target genes. We have recently reported that integrin-linked kinase (ILK) mediates pro-inflammatory cytokine TNF-α production by modulating phosphorylation of NF-κB p65 at Ser536 in response to pathogenic microorganisms [42]. According to our observations, ILK is required for an alternative activation of NF-κB through p65 Ser536 phosphorylation, as the classical activation of NF-κB involving IκBα degradation and nuclear translocation of p65 remains unaffected by ILK inhibition. ILK mediates p65 Ser536 phosphorylation in response to both LPS and *H. pylori* infection; for the latter this event is dependent on the PI3K/Akt pathway (Figure 2).

RelA is also phosphorylated at multiple serine residues in the transactivation domain of p65. To date, 12 putative p65 phosphorylation sites have been identified [43]. Among them, five sites (Ser205, Ser276, Ser281, Ser311 and Thr264) are located in the N-terminal Rel homology domain (RHD) and the remaining seven (Ser468, Ser529, Ser535, Ser536, Ser547, Thr435, and Thr505) are at the C-terminal transactivation domain. RelA is also post-translationally modified through methylation at Lys-47 by Set9 methyltransferase and this modification is required for the selective activation of NF-κB target genes [44]. The methylation at Lys-47 of RelA is required for the enhanced stability of RelA binding to select recognition sites, facilitating the transcriptional activation of genes that would otherwise remain inactive. RelA is also acetylated by HATs (such as p300 and CBP) at multiple residues (such as Lys-122, Lys-123, Lys-218, Lys-221, and Lys-310) [45,46]. Since lysine residues at 122 and 123 are important for high affinity binding of p65 to κB-DNA, acetylation of p65 at lysine 122/123 represses it transcriptional activity [46]. Interestingly, acetylation of Lys211 and Lys310 augments both DNA binding and transactivation capacity of RelA by reducing its interaction with IκBα [47].

The specificity of NF-κB-mediated transcriptional activation of pro-inflammatory genes can be influenced further by dimers formed within the five members of the NF-κB family. It has been proposed that these dimers may regulate different sets of genes through their ability to interact with unique sets of regulatory proteins [15,48]. Despite a significant redundancy in transcriptional activities among the dimers, the understanding of the dimer-specific functions is still far from being complete [48]. It has been proposed that the specificity of each dimer formation is dependent on both the signaling pathway and physiological conditions. The activation kinetics of NF-κB transcriptional function also provides a vital contribution to the specificity of selective gene activation. The timing of NF-κB nuclear translocation and the export from nucleus followed by its inactivation, as well as the duration of NF-κB activation, vary significantly depending on specificity of the stimulus. Interestingly, during NF-κB activation, the association of NF-κB with its target genes also proceeds with variable kinetics. For example, using chromatic immunoprecipitation (ChIP), it was shown that the binding of NF-κB to the *Cxcl2* and *Nfkbia* promoters took place soon after it entered the nucleus, whereas the binding to other promoters was delayed significantly [49]. This variability leads to substantial differences in the activation kinetics and expression pattern of selective genes in response to a stimulus [50].

## 5. Ubiquitination of Inflammatory Signaling Complexes

**Figure 3:**
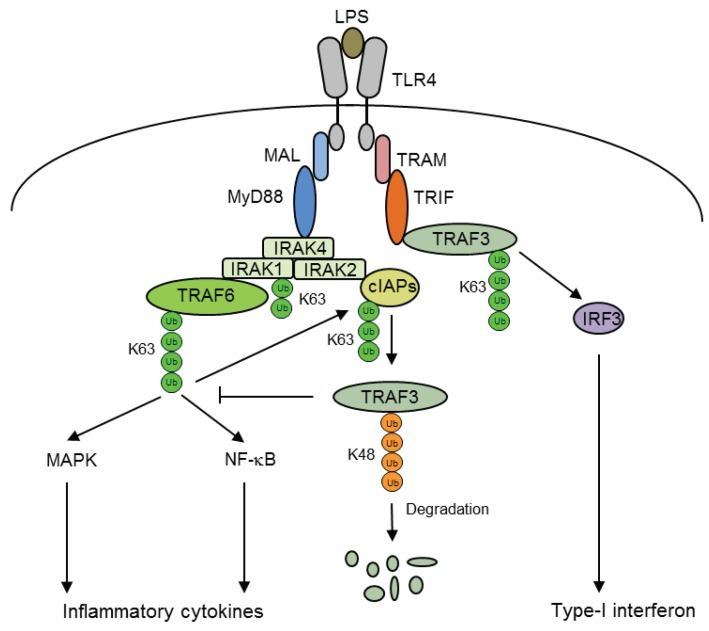
Ubiquitination-mediated signaling cascades in TLR4-induced gene activation. In response to LPS, cellular inhibitor of apoptosis protein (cIAPs) are activated via K63-linked ubiquitination by TNF receptor-associated factor (TRAF) 6. Activated cIAPs target TRAF3 for proteasomal degradation via K48-linked ubiquitination, which is required for LPS-mediated activation of pro-inflammatory cytokines. In MyD88-independent signaling, TRAF3 is activated via K63-linked ubiquitination by TRIF, which is required for IRF3 activation and the IFN response.

The inflammatory gene transcription program is tightly regulated by the assembly of an ubiquitin (Ub)-dependent signaling complex in which E3 ubiquitin ligases along with E1 ubiquitin-activating and E2 ubiquitin-conjugating enzymes attach ubiquitin molecule(s) to a lysine of the substrate protein. Depending on the nature of the ubiquitin chains linked to the substrate protein, ubiquitination serves critical signaling roles. For example, Lys63 (K63)-linked chains allow assembly of protein complexes that lead to the activation of kinases and Lys48 (K48)-linked chains target substrates for proteasomal degradation [51]. Following ligand binding to TLRs, IRAK4 is recruited to MyD88 and forms a complex with kinases IRAK1 and IRAK2, the E3 ubiquitin ligase TRAF6 and the E2 ubiquitin-conjugating enzyme 13 (UBC13). TRAF6 and UBC13 catalyse the formation of K63-linked polyubiquitin chains on both TRAF6 and IRAK1, which subsequently induce MAPK and NF-κB pathways by activating the TAK1/TAB1/TAB2/TAB2, and IKK complex, respectively [52] (Figure 3).

The outcomes of the inflammatory signaling pathways are profoundly dictated by differential ubiquitination-mediated regulation. In response to LPS stimulation, the selective transcriptional activation of either inflammatory genes or type-1 IFNs is mediated by the alternative ubiquitination modes of the adaptor protein TRAF3 [53]. Being incorporated in both MyD88- and TRIF-assembled multi-protein complexes during LPS stimulation, TRAF3 plays unique roles as a negative regulator of the MyD88-dependent inflammatory response, as well as a positive regulator of the TRIF-dependent IFN response. In the MyD88-dependent signaling complex, TRAF3 undergoes K48-linked ubiquitination by cellular inhibitor of apoptosis protein 1 (cIAP1) and cIAP2, which are activated by TRAF6 via K63-linked ubiquitination and act as K48-specific E3 ligases for TRAF3. Following degradation of TRAF3, the MyD88-associated signaling complex translocates to the cytoplasm, where it stimulates MAPK activation and production of inflammatory cytokines. In contrast, TRIF-mediated signaling triggers TRAF3 self-ubiquitination through K63-linked ubiquitination, which leads to IRF3 activation and the IFN response [53] (Figure 3).

**Figure 4:**
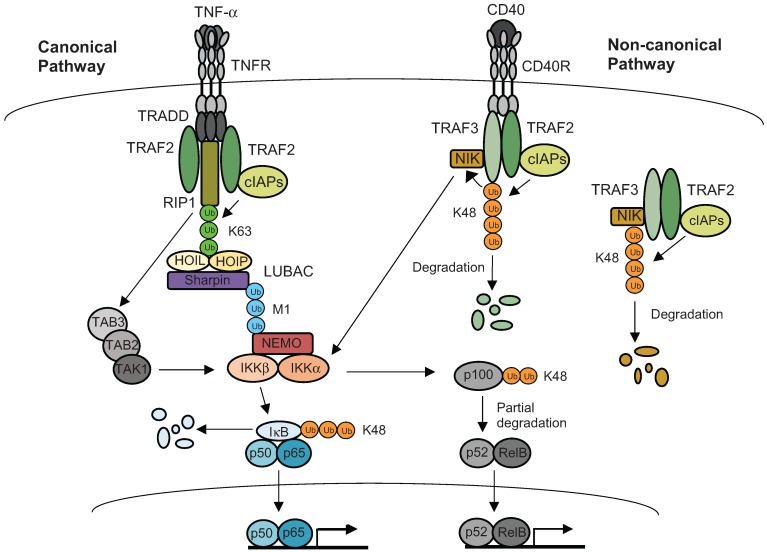
Ubiquitination-mediated signaling cascades in canonical and non-canonical NF-κB pathways. In canonical pathway, binding of TNF-α to TNFR1 triggers recruitment of the adaptor protein TRADD, which stimulates the formation of a complex consisting of TRAF2, RIP1, cIAP1 and cIAP2. Additionally, cIAPs promote K63 linkage-mediated ubiquitination of RIP1, which in turn leads to the recruitment of LUBAC. LUBAC results in stabilization of the complex by further ubiquitination, thereby providing the docking sites of TAB2/TAB3/TAK1 and IKK complexes, as well as their activation. The activation of IKKβ leads to K48-linked polyubiquitination and proteasomal degradation of IκB, thus facilitating liberation of p65/p50. During non-canonical activation (e.g., CD40 ligation), cIAP1/2 are recruited to receptor complexes by TRAF2 where they target TRAF3 for K48-linked ubiquitination and proteasomal destruction, thereby facilitating the release of NIK. The accumulated NIK phosphorylates IKKα, which in turn leads to a partial degradation of p100 to p52, facilitating nuclear translocation of RelB:p52 dimer. Under resting conditions, NIK is consistently targeted by cIAPs for K48-linked ubiquitylation and subsequent degradation.

NF-κB signal transduction is strongly modulated by cIAP1 and cIAP2 through their activity as E3 ligases. Best studied in TNF family-induced NF-κB signaling, cIAPs are critical regulators of stimulus-dependent activation of the canonical as well as constitutive suppression of the non-canonical NF-κB pathways [52]. The activated TNF-receptor (TNFR)-mediated signaling complex contains the adaptor proteins TRADD, Sam68, TRAF2 and TRAF5; the E3 ligases cIAP1 and cIAP2; and the protein kinase RIPK1. Following recruitment by TRAF2 to the complex, cIAPs promote polyubiquitination of RIPK1. These ubiquitination events provide a platform for the subsequent recruitment of the Linear UB chain Assembly Complex (LUBAC, composed of HOIL/HOIP/Sharpin), and the kinase complexes TAK1/TAB2/TAB3, and IKK (composed of NEMO/IKKα/ IKKβ). Once recruited, LUBAC enhances the stability of the complex by modifying NEMO and RIPK1 with M1-linked Ub chains, thus allowing the formation of fully functional signaling complexes and the activation of IKKβ kinase activity. After activation, IKKβ phosphorylates IκB, thereby targeting IκB for ubiquitination and proteasomal degradation, to liberate the p50/RelA dimer and activate the canonical NF-κB pathway. Apart from inducing RIPK1 degradation, c-IAPs were also shown to be indispensable for the recruitment of IKKβ, NEMO and HOIP to TNFR. In addition, c-IAPs are also required for MAPKs JNK and p38 signaling during TNFR stimulation, thus making a critical impact on the transcription of pro-inflammatory genes [54] (Figure 4).

In non-canonical NF-κB signaling, cIAPs are responsible for the ubiquitination and subsequent degradation of NF-κB-inducing kinase (NIK), the key regulator of non-canonical NF-κB signaling. Under unstimulated conditions, non-canonical NF-κB signaling is normally suppressed because of constitutive proteasomal degradation of NIK, mediated by a complex consisting of TRAF2, TRAF3 and cIAPs. Through a heterodimeric bond with TRAF3 that directly binds to NIK, TRAF2 brings cIAP proteins in the proximity of NIK, thus promoting K48-linked ubiquitination of NIK for its degradation. Following stimulation of any one of several TRAF3-binding TNF superfamily receptors (BAFF, CD40L or TWEAK), the cytoplasmic TRAF3-TRAF2-cIAP complex is disrupted by the membrane recruitment and degradation of its components. The depletion of the TRAF3-TRAF2-cIAP E3 complex results in stabilization of NIK, which subsequently phosphorylates IKKα and the NF-κB precursor p100. Activated IKKα homodimers phosphorylate additional residues in p100, which triggers its partial degradation to generate the p52 form. The p52 fragment dimerizes with RelB and translocates to the nucleus where it binds the promoter regions of NF-κB-dependent genes to activate their transcription [55,56] (Figure 4).

## 6. Selective Regulation by Chromatin Structures

The selective transcription of pro-inflammatory genes involving differential binding of transcription factors to specific DNA sequences is largely influenced by chromatic structures that make up chromosomes. The fundamental subunits of chromatin are nucleosomes, which consist of a segment of DNA wrapped around a histone octamer containing two copies each of four histone proteins, H1A, H2B, H3, and H4 [57]. The structural unit of chromatin also contains linker histones and other non-histone proteins. Based on sensitivity analyses of cell nuclei to cleavage by nucleases, it was demonstrated decades ago that the accessibility of transcription factors and RNA polymerase to given promoters was dependent on chromatin structures [58,59]. It was proposed that, upon a specific stimulation, chromatin at selected genes undergoes conformational change to become accessible to transcription factor binding in order to facilitate the transcription of those genes. These conformational changes, known as nucleosome remodeling, are mediated by the dissociation of the chromatin structures through removal or translocation of nucleosomes [60]. The selective regulation of pro-inflammatory genes by chromatin structures was also reflected in NF-κB-mediated pro-inflammatory gene activation during LPS stimulation. It has been demonstrated that in LPS-stimulated macrophages, NF-κB binds to its target genes in the nuclei with variable kinetics [49]. It has been proposed that the binding of NF-κB to some of its target genes is delayed by the nucleosome barrier until additional remodeling factors become available to facilitate NF-κB access to those genes.

The first evidence for a direct role for chromatins in transcriptional regulation came from an observation in *S. cerevisiae* that the acetylation of histone H3 is mediated by a transcriptional coactivator, Gcn5 [61]. The SWI/SNF complex, known as an important regulator of selective yeast genes, was also demonstrated earlier as a critical regulator of nucleosome conformation by a number of studies [62,63,64]. The SWI/SNF complex was also shown to be recruited to chromatin immediately after the activation of T cells, and the recruitment of the SWI/SNF complex was associated with a global decondensation of the chromatin, which is required for T cell activation [65]. Based on shRNA-mediated depletion of core subunits of the mammalian SWI/SNF complex, it has been shown that in response to LPS almost all secondary response genes are dependent on the SWI/SNF complex, whereas the majority of the primary response genes are activated in a SWI/SNF-independent manner [66]. The promoters of SWI/SNF-independent genes were shown to be fully accessible to nuclease cleavage regardless of any stimulation, but SWI/SNF-dependent genes showed accessibility only after stimulation suggesting a need to overcome nucleosome barriers prior to gene activation. Interestingly, SWI/SNF-independent genes were also shown to be assembled in a pre-active state with high histone acetylation and histone H3K4 trimethylation prior to stimulation and therefore the activation of these genes proceeds without any requirement for the SWI/SNF complex [66,67]. SWI/SNF-independent genes were also shown to be associated with CpG island promoters, whereas SWI/SNF-dependent genes contain low CpG island promoters. As nucleosomes assembled on CpG island promoters are less stable, CpG island promoters lack a nucleosome barrier, whereas the low CpG promoters are assembled into stable nucleosomes with a requirement for nucleosome remodeling before activation [67].

The activation of SWI/SNF-dependent genes through nuclear remodeling suggests a model in which the recruitment of the SWI/SNF complex to its target genes is dependent on stimulus-specific primary response gene products such as transcription factors. In accord with this model, a substantial number of SWI/SNF-dependent genes possess consensus IRF3 binding sites on their promoters and the activation of these genes in response to LPS stimulation was shown to be dependent on IRF3 binding to their promoters [67]. Furthermore, LPS-induced nucleosome remodeling at these promoters was completely eliminated in macrophages from IRF3 knockout mice, confirming a critical role for IRF3 in nucleosome remodeling. Nucleosome remodeling is in fact a more critical step towards selective regulation of gene activation than cooperative binding of transcription factors to the promoters of these genes. This view was supported by a study on the IRF3-dependent IFN-β promoter, which forms a stable complex of IRF3, NF-κB and ATF2/c-Jun upon stimulation, showing a requirement for all three transcription factors [68]. According to this study, following the elimination of the nucleosome barrier through artificial positioning of the nucleosome, the IFN-β promoter was fully activated by NF-κB and ATF2/c-Jun binding only in the absence of IRF3. This study further consolidates a critical role for nucleosome barriers in restricting selective activation of genes.

**Figure 5:**
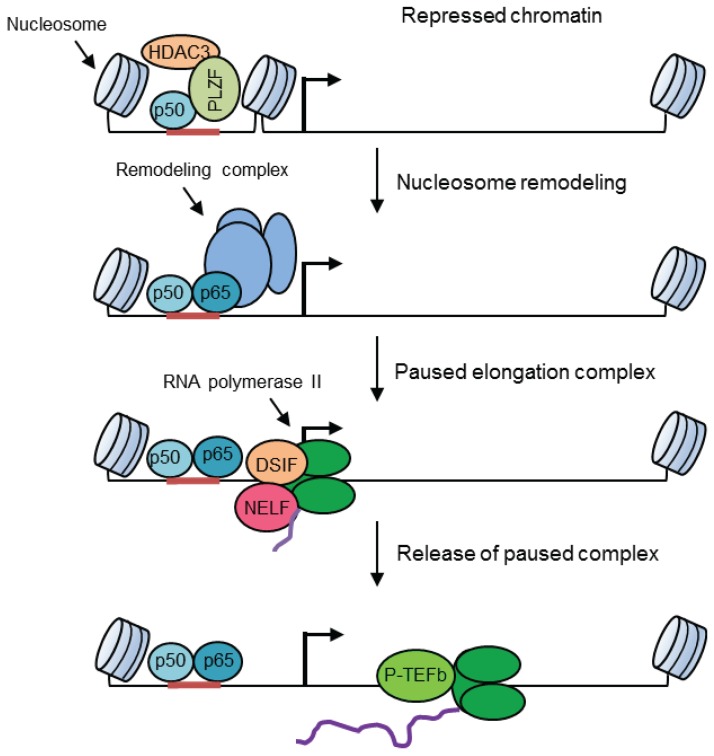
A schematic diagram showing a repressed chromatin state, nucleosome remodeling, paused elongation complex and signal-dependent pause release for the initiation of productive elongation. A transcriptionally repressed chromatin state is shown in which PLZF-mediated repressor complex comprising HDAC3 and p50 inhibits the formation of an active elongation complex. A remodeling complex induces transcription factor binding by removing nucleosomes, thus providing access of the transcription machinery to the promoter. Pausing during early elongation is facilitated by DSIF and NELF. The signal-dependent recruitment of P-TEFb results in pause release via the dissociation of DSIF and NELF from the elongation complex.

The transcriptional regulator PLZF is a critical co-factor that limits NF-κB-mediated inflammatory responses by maintaining a transcriptionally repressed chromatin state. PLZF stabilizes a repressor complex consisting of HDAC3 and the NF-κB p50 subunit to the promoters of select genes (Figure 5), thereby limiting the formation of the active transcription complexes by restricting the binding of p65 and p300 to RNA polymerase II. The assembly of the PLZF-mediated repressor complex is mediated by the acetylation of PLZF by HAT1, which is activated by CaMK2 [22,23], suggesting an involvement of the calcium-signaling pathway in chromatin modifications.

Pausing of the early elongation complex has also been described for several genes in which the synthesis of a short RNA-by-RNA polymerase II is paused until an appropriate signal for productive elongation appears [69]. This phenomenon is mediated by pause-inducing factors, DRB sensitivity-inducing factor (DSIF) and negative elongation factor (NELF) [70]. Upon a stimulation for productive elongation, the release of paused polymerase is facilitated by the kinase activity of the positive transcription elongation factor b (P-TEFb), which phosphorylates DSIF and/or NELF as well as the C-terminal domain (CTD) of the largest regulatory subunit of the polymerase, creating a binding platform for various RNA processing and chromatin-modifying factors for productive elongation (Figure 5). The recruitment of P-TEFb to promoters is also a complex process involving factors such as NF-κB [71], Mediator complex [72], and bromodomain and extraterminal (BET) proteins that bind acetylated histones [73] (Figure 5).

The transcription of a target gene is also positively influenced by enhancers located at a considerable distance. It has been suggested that that enhancers facilitate recruitment of the transcription machinery to the target promoter via DNA loops, bringing enhancer to the close proximity of the promoter [74,75]. The transcription activity at enhancers also plays a critical role for several cytokines such as IL-4 and IL-12b [76,77]. In fact, a mechanistic link between noncoding enhancer RNA (eRNA) and the corresponding promoter-associated nascent mRNA was first proposed by an observation that the kinetics of both RNA synthesis are very similar upon stimulation [78]. Although the mechanisms underlying enhancer function are still poorly defined, recent studies suggest that noncoding eRNAs facilitate transcription activation of neighbouring genes by modulating chromatin organization or promoter-enhancer close proximity via DNA loop [79,80].

The concept of nucleosome remodeling for the activation of selective genes is far from being completely understood, as the primary gene products required for remodeling remain largely unknown. Moreover, the potential involvement of additional regulatory layers in chromatin structures and remodeling cannot be ignored, as the activation of the SWI/SNF complex following its recruitment to target genes upon LPS stimulation was shown to be dependent on a calcium signaling pathway [81].

Recent observations suggest that the differential kinetics of gene activation cannot be explained solely based on the promoter accessibility [69,82,83]. There is no mechanistic link that exists between activation kinetics and CpG content or presence of paused polymerase and open chromatin architecture, suggesting that the additional factors are involved in the precise regulation of transcriptional gene activation. Nevertheless, the specific patterns of inflammatory gene expression are not only defined by signal-dependent factors, but also signal-independent factors associated with the chromatin over the course of development [69].

## 7. Additional Effects on Pro-Inflammatory Gene Transcription

The selective transcription of pro-inflammatory genes is predominantly dictated by both the specificity of the signaling cascades, ubiquitination, and transcription factors, and the nature of the chromatin states of these genes as discussed above. According to the current literature, inflammatory gene transcription is also subjected to the consequences of various molecular features and/or physiological events at the cellular level. Some of these influences are briefly discussed below.

### 7.1. Epigenetic Markers

Over the last decade, an array of epigenetic markers has emerged as defining features for active or repressed chromatin states, thereby influencing transcriptional activation. For example, the recruitment of NF-κB to the promoter regions of many pro-inflammatory genes is promoted by the acetylation of histone H3 at these promoters [84]. Acetylation of histones promotes transcription of genes by facilitating the relaxation of chromatin structures whereas the HDAC activity is associated with the repression of these genes [85]. In accord with an essential role of histone acetylation for transcriptional activation, PLZF-deficient cells display an increased level of histone marks such as acetylated lysine 27 (H3K27ac) and trimethylated lysine residue 4 (H3K4me3), which are a distinctive feature of active transcription [22]. On the other hand, histone methylation can act either as a repressor or as an activator of transcription of genes, depending on the type of methylation [85]. For example, tri-methylation of histone H3 on lysine 9 (H3K9me3) is associated with transcriptional repression and is present at the promoters of some but not all inducible genes in unstimulated cells. H3K9me3 was observed at the promoters of IL12B, CCL19 and CCL22 in unstimulated human dendritic cells and was lost immediately after LPS stimulation [86]. This model also suggests the recruitment of specific histone demethylase to facilitate demethylation during stimulation, but unfortunately the majority of these histone demethylases remain to be identified. Interestingly, a histone demethylase, Jmjd3, was shown to be associated with a subset of genes in unstimulated macrophages [87,88]. In contrast, tri-methylations of histone 3 on lysine 4 and 36 (H3K4me3 and H3K36me3) activate transcription through the relaxation of the chromatin structures [89]. The methylation on DNA sequence also serves as a critical regulator of gene transcription. The methylated CpG islands at the noncoding regions of the genome were reported as repressors of transcriptional activation [90]. The changes in DNA methylation, such as hypomethylation, have significant impact on transcription through their association with chromosome instability and activation of transposable elements in human cancers [91]. For example, DNA hypomethylation was observed at the TLR2 promoter in cystic fibrosis epithelial cells during the inflammatory response to bacterial peptidoglycan [92]. Certain post-translational histone modifications such as monomethylation of histone H3, lysine 4 (H3K4me1), and acetylation of histone H3 lysine 27 (H3K27Ac) are also associated enhancer regions [93,94].

### 7.2. Developmental Events

The chromatin structures at the promoters of inducible genes in resting cells, and the patterns of the subsequent transcriptional activation of these genes upon stimulation vary significantly from one cell type to another in eukaryotic organisms. These cell-type-specific variations indicate the existence of multiple transcriptional mechanisms operating in a developmental-stage-specific manner. In fact, this view was supported by several reports demonstrating that the cell-type-specific variations in chromatin structures and transcriptional mechanisms were established early in development long before these genes are activated [95,96,97]. Consistent with the view that the selective regulation of inducible genes is dictated by developmental events, it has been shown that LPS-stimulated IL-6 expression in mouse macrophages requires nucleosome remodeling by SWI/SNF complexes due to an inaccessible chromatin structure at the IL-6 promoter, but in fibroblasts IL-6 expression proceeds in a SWI/SNF-independent manner due to an open chromatin structure at the promoter [67]. However, the mechanisms of chromatin remodeling that underlie developmental variations in transcriptional activation of genes encoding inflammatory mediators remain to be fully characterized.

### 7.3. Physiological Relevance

It would be hard to imagine that the mechanistic diversity in selective inducible gene activations as discussed above operates without any physiological relevance. For example, macrophages are prominent for their ability to fight against infectious agents, as well as for tissue healing and therefore they remain abundant at the sites of infections or tissue injuries. Compared to other responsive cell types such as fibroblasts, macrophages are mobile and can easily accumulate as needed. Therefore, the production of pro-inflammatory cytokines by macrophages at these sites needs to be precisely controlled with additional regulatory layers, not only to cope with the presence of a plentiful supply of inflammatory stimuli associated with these sites, but also to avoid excessive tissue injuries. As a result, SWI/SNF-dependent and SWI/SNF-independent expression of IL-6 in macrophages and fibroblasts, respectively, upon LPS stimulation [67], is likely physiologically relevant. Another example of physiological relevance to the selective regulation of inflammatory cytokine production is the tolerance of macrophages to repetitive stimulation by LPS. During repetitive stimulation, the expression of pro-inflammatory genes is selectively repressed to avoid tissue injuries, while the expression of anti-microbial genes remains uninterrupted [98]. Besides these phenomena, the correlation between the mechanistic diversity in selective transcriptional activation of pro-inflammatory genes and physiological regulation remains to be fully defined.

### 7.4. Interplay between Signaling Pathways

The induction of selective transcriptional activation of pro-inflammatory genes has been considered to be driven by inflammatory stimuli through a common set of transcription factors such as NF-κB, AP-1, CREB, and others, as mentioned above. However, a number of recent observations have revealed that pro-inflammatory signal transduction pathways are far more diverse than previously realized, indicating an involvement of multiple signaling pathways in selective transcription of pro-inflammatory genes. As mentioned above, a calcium signaling pathway plays a critical role in activating the SWI/SNF complex following its recruitment to its target genes [81]. The transcription factor p53 is well known for its roles in DNA repair and in the regulation of a wide variety of genes involved in apoptosis. Due to its role in preventing genomic mutation and cancer, p53 is described as a tumour suppressor protein [99]. Recent observations have demonstrated that p53 is also required for the up-regulation of TLR-dependent pro-inflammatory cytokines [100]. It has also been revealed that both p53 and NF-κB co-regulate the transcription of pro-inflammatory genes in human monocytes and macrophages [101]. Further studies are needed to uncover a more comprehensive understanding of the roles and interactions of multiple signaling pathways in selective transcription of pro-inflammatory genes.

## 8. Conclusions and Future Perspectives

A great depth of knowledge has been gained about the selective transcription of pro-inflammatory genes since the discovery of NF-κB more than 25 years ago [14]. Using RNA-seq analysis as a method to monitor nascent RNA transcripts, it has been revealed that within each selectively activated group, genes are not perfectly co-regulated with respect to transcription kinetics [82]. This observation suggests that each inducible gene has its unique transcriptional kinetics within the same group of selectively activated genes, even upon exposure to the same stimulus. The more we learn about the pro-inflammatory gene transcription, the more diverse the underlying mechanisms seem to be. Apart from the diversity in signaling pathways involving an array of transcription factors to regulate pro-inflammatory gene expression, the field has been rapidly evolving over the last decade through the identification of new molecules playing critical roles in ubiquitination, chromatin structures, and epigenetic features. Despite considerable advances made over the years, there is still a lot to be learned and revealed about the pro-inflammatory transcription field. Many of the regulatory proteins involved in the post-translational modifications of signaling components, transcription factors and in the epigenetic alterations of chromatins during inflammatory stimulation remain to be fully characterized. Others have also have identified ILK as a critical regulator of NF-κB activation, as have we [42,102,103,104,105,106,107]. We have demonstrated ILK-dependent phosphorylation of p65 at Ser536, activating NF-κB and inducing TNF-α expression in response to both LPS stimulation and *H. pylori* infection [42]. Since ILK has been known as a focal adhesion kinase, our observation has not only introduced a new regulatory player to this field, but has also further emphasized the critical involvement of integrins and other signaling molecules of focal adhesions in both NF-κB activation and pro-inflammatory signal transduction, in accordance with published reports [108,109,110,111,112,113,114,115]. In addition to NF-κB activation, ILK has also been implicated in the MAPK signaling pathway for its critical roles in various pathological and physiological functions, such as non-small cell lung cancer (NSCLC) cell growth [116], osteoblast differentiation [117], bladder cancer cell migration [118], and rheumatoid synovial cell survival [119]. The MAP3K ASK1 signaling is also regulated by glycogen synthase kinase 3β (GSK3β) [120], which is also known as a downstream target of ILK. These studies have strongly emphasized ILK as an integrated signaling molecule for both NF-κB and MAPK activation, leading to inflammatory gene transcription. Only further investigation can provide important insights into inflammatory transcriptional mechanisms, and holds the promise of uncovering exciting new avenues of inflammatory gene regulation.

## References

[1] Medzhitov R. (2010). Inflammation 2010: New adventures of an old flame. Cell.

[2] Latz E., Xiao T.S., Stutz A. (2013). Activation and regulation of the inflammasomes. Nat. Rev. Immunol..

[3] Kumar V., Carey M., Robbins S.L. (2003). Robbins Basic Pathology.

[4] Majno G., Joris I. (2004). Cells, Tissues, and Diseases.

[5] Meroni P.L., Valentini G., Ayala F., Cattaneo A., Valesini G. (2015). New strategies to address the pharmacodynamics and pharmacokinetics of tumor necrosis factor (TNF) inhibitors: A systematic analysis. Autoimmun. Rev..

[6] Kodama S., Davis M., Faustman D.L. (2005). The therapeutic potential of tumor necrosis factor for autoimmune disease: A mechanistically based hypothesis. Cell. Mol. Life Sci..

[7] Akira S., Uematsu S., Takeuchi O. (2006). Pathogen recognition and innate immunity. Cell.

[8] Yamamoto K.R., Alberts B.M. (1976). Steroid receptors: Elements for modulation of eukaryotic transcription. Annu. Rev. Biochem..

[9] Herschman H.R. (1991). Primary response genes induced by growth factors and tumor promoters. Annu. Rev. Biochem..

[10] Smale S.T. (2010). Selective transcription in response to an inflammatory stimulus. Cell.

[11] Kawai T., Akira S. (2010). The role of pattern-recognition receptors in innate immunity: Update on toll-like receptors. Nat. Immunol..

[12] McGettrick A.F., O’Neill L.A. (2010). Regulators of TLR4 signaling by endotoxins. Subcell. Biochem..

[13] Newton K., Dixit V.M. (2012). Signaling in innate immunity and inflammation. Cold Spring Harb. Perspect. Biol..

[14] Sen R., Baltimore D. (1986). Inducibility of kappa immunoglobulin enhancer-binding protein NF-kappaB by a posttranslational mechanism. Cell.

[15] Ghosh S., May M.J., Kopp E.B. (1998). NF-kappa B and Rel proteins: Evolutionarily conserved mediators of immune responses. Annu. Rev. Immunol..

[16] Hoffmann A., Natoli G., Ghosh G. (2006). Transcriptional regulation via the NF-kappaB signaling module. Oncogene.

[17] Ghosh S., Karin M. (2002). Missing pieces in the NF-kappaB puzzle. Cell.

[18] Vallabhapurapu S., Karin M. (2009). Regulation and function of NF-kappaB transcription factors in the immune system. Annu Rev. Immunol..

[19] Ahmed A.U. (2011). An overview of inflammation: Mechanism and consequences. Front. Biol..

[20] Sun S.C. (2012). The noncanonical NF-kappaB pathway. Immunol. Rev..

[21] Arthur J.S., Ley S.C. (2013). Mitogen-activated protein kinases in innate immunity. Nat. Rev. Immunol..

[22] Sadler A.J., Rossello F.J., Yu L., Deane J.A., Yuan X., Wang D., Irving A.T., Kaparakis-Liaskos M., Gantier M.P., Ying H. (2015). BTB-ZF transcriptional regulator PLZF modifies chromatin to restrain inflammatory signaling programs. Proc. Natl. Acad. Sci. USA.

[23] Sadler A.J., Suliman B.A., Yu L., Yuan X., Wang D., Irving A.T., Sarvestani S.T., Banerjee A., Mansell A.S., Liu J.P. (2015). The acetyltransferase hat1 moderates the NF-kappaB response by regulating the transcription factor PLZF. Nat. Commun..

[24] Carey M.F., Peterson C.L., Smale S.T. (2009). Transcriptional Regulation in Eukaryotes: Concepts, Strategies, and Techniques.

[25] Thanos D., Maniatis T. (1995). Virus induction of human IFN beta gene expression requires the assembly of an enhanceosome. Cell.

[26] Munshi N., Agalioti T., Lomvardas S., Merika M., Chen G., Thanos D. (2001). Coordination of a transcriptional switch by HMGI(Y) acetylation. Science.

[27] Bosisio D., Marazzi I., Agresti A., Shimizu N., Bianchi M.E., Natoli G. (2006). A hyper-dynamic equilibrium between promoter-bound and nucleoplasmic dimers controls NF-kappaB-dependent gene activity. EMBO J..

[28] Hager G.L., McNally J.G., Misteli T. (2009). Transcription dynamics. Mol. Cell.

[29] Panne D., Maniatis T., Harrison S.C. (2004). Crystal structure of ATF-2/c-jun and IRF-3 bound to the interferon-beta enhancer. EMBO J..

[30] Panne D., Maniatis T., Harrison S.C. (2007). An atomic model of the interferon-beta enhanceosome. Cell.

[31] Wan F., Lenardo M.J. (2009). Specification of DNA binding activity of NF-kappaB proteins. Cold Spring Harb. Perspect. Biol..

[32] Oeckinghaus A., Ghosh S. (2009). The NF-kappaB family of transcription factors and its regulation. Cold Spring Harb. Perspect. Biol..

[33] Zhong H., Voll R.E., Ghosh S. (1998). Phosphorylation of NF-kappa B p65 by Pka stimulates transcriptional activity by promoting a novel bivalent interaction with the coactivator Cbp/p300. Mol. Cell.

[34] Zhong H., May M.J., Jimi E., Ghosh S. (2002). The phosphorylation status of nuclear NF-kappaB determines its association with Cbp/p300 or Hdac-1. Mol. Cell.

[35] Dong J., Jimi E., Zhong H., Hayden M.S., Ghosh S. (2008). Repression of gene expression by unphosphorylated NF-kappaB p65 through epigenetic mechanisms. Genes Dev..

[36] Sakurai H., Chiba H., Miyoshi H., Sugita T., Toriumi W. (1999). Ikappab kinases phosphorylate NF-kappaB p65 subunit on serine 536 in the transactivation domain. J. Biol. Chem..

[37] Yang F., Tang E., Guan K., Wang C.Y. (2003). IKK beta plays an essential role in the phosphorylation of RelA/p65 on serine 536 induced by lipopolysaccharide. J. Immunol..

[38] Douillette A., Bibeau-Poirier A., Gravel S.P., Clement J.F., Chenard V., Moreau P., Servant M.J. (2006). The proinflammatory actions of angiotensin II are dependent on p65 phosphorylation by the IkappaB kinase complex. J. Biol. Chem..

[39] Mandrekar P., Jeliazkova V., Catalano D., Szabo G. (2007). Acute alcohol exposure exerts anti-inflammatory effects by inhibiting IkappaB kinase activity and p65 phosphorylation in human monocytes. J. Immunol..

[40] Nicholas C., Batra S., Vargo M.A., Voss O.H., Gavrilin M.A., Wewers M.D., Guttridge D.C., Grotewold E., Doseff A.I. (2007). Apigenin blocks lipopolysaccharide-induced lethality *in vivo* and proinflammatory cytokines expression by inactivating NF-kappaB through the suppression of p65 phosphorylation. J. Immunol..

[41] Sasaki C.Y., Barberi T.J., Ghosh P., Longo D.L. (2005). Phosphorylation of RelA/p65 on serine 536 defines an I{kappa}B{alpha}-independent NF-{kappa}B pathway. J. Biol. Chem..

[42] Ahmed A.U., Sarvestani S.T., Gantier M.P., Williams B.R., Hannigan G.E. (2014). Integrin-linked kinase modulates lipopolysaccharide- and helicobacter pylori-induced nuclear factor kappaB-activated tumor necrosis factor-alpha production via regulation of p65 serine 536 phosphorylation. J. Biol. Chem..

[43] Hochrainer K., Racchumi G., Anrather J. (2013). Site-specific phosphorylation of the p65 protein subunit mediates selective gene expression by differential NF-kappaB and RNA polymerase II promoter recruitment. J. Biol. Chem..

[44] Ea C.K., Baltimore D. (2009). Regulation of NF-kappaB activity through lysine monomethylation of p65. Proc. Natl. Acad. Sci. USA.

[45] Chen L.F., Greene W.C. (2003). Regulation of distinct biological activities of the NF-kappaB transcription factor complex by acetylation. J. Mol. Med..

[46] Kiernan R., Bres V., Ng R.W., Coudart M.P., el Messaoudi S., Sardet C., Jin D.Y., Emiliani S., Benkirane M. (2003). Post-activation turn-off of NF-kappa B-dependent transcription is regulated by acetylation of p65. J. Biol. Chem..

[47] Chen L.F., Mu Y., Greene W.C. (2002). Acetylation of rela at discrete sites regulates distinct nuclear functions of NF-kappaB. EMBO J..

[48] Smale S.T. (2012). Dimer-specific regulatory mechanisms within the NF-kappaB family of transcription factors. Immunol. Rev..

[49] Saccani S., Pantano S., Natoli G. (2001). Two waves of nuclear factor kappaB recruitment to target promoters. J. Exp. Med..

[50] Smale S.T. (2011). Hierarchies of NF-kappaB target-gene regulation. Nat. Immunol..

[51] Corn J.E., Vucic D. (2014). Ubiquitin in inflammation: The right linkage makes all the difference. Nat. Struct. Mol. Biol..

[52] Darding M., Meier P. (2012). IAPs: Guardians of RIPK1. Cell Death Differ..

[53] Tseng P.H., Matsuzawa A., Zhang W., Mino T., Vignali D.A., Karin M. (2010). Different modes of ubiquitination of the adaptor TRAF3 selectively activate the expression of type I interferons and proinflammatory cytokines. Nat. Immunol..

[54] Varfolomeev E., Goncharov T., Maecker H., Zobel K., Komuves L.G., Deshayes K., Vucic D. (2012). Cellular inhibitors of apoptosis are global regulators of NF-kappaB and mapk activation by members of the TNF family of receptors. Sci. Signal..

[55] De Almagro M.C., Vucic D. (2012). The inhibitor of apoptosis (Iap) proteins are critical regulators of signaling pathways and targets for anti-cancer therapy. Exp. Oncol..

[56] Silke J., Vucic D. (2014). IAP family of cell death and signaling regulators. Methods Enzymol..

[57] Luger K., Mader A.W., Richmond R.K., Sargent D.F., Richmond T.J. (1997). Crystal structure of the nucleosome core particle at 2.8 A resolution. Nature.

[58] Weintraub H., Groudine M. (1976). Chromosomal subunits in active genes have an altered conformation. Science.

[59] Wu C., Bingham P.M., Livak K.J., Holmgren R., Elgin S.C. (1979). The chromatin structure of specific genes: I. Evidence for higher order domains of defined DNA sequence. Cell.

[60] Clapier C.R., Cairns B.R. (2009). The biology of chromatin remodeling complexes. Annu. Rev. Biochem..

[61] Brownell J.E., Zhou J., Ranalli T., Kobayashi R., Edmondson D.G., Roth S.Y., Allis C.D. (1996). Tetrahymena histone acetyltransferase A: A homolog to yeast Gcn5p linking histone acetylation to gene activation. Cell.

[62] Cote J., Quinn J., Workman J.L., Peterson C.L. (1994). Stimulation of gal4 derivative binding to nucleosomal DNA by the yeast SWI/SNF complex. Science.

[63] Imbalzano A.N., Kwon H., Green M.R., Kingston R.E. (1994). Facilitated binding of TATA-binding protein to nucleosomal DNA. Nature.

[64] Kwon H., Imbalzano A.N., Khavari P.A., Kingston R.E., Green M.R. (1994). Nucleosome disruption and enhancement of activator binding by a human SW1/SNF complex. Nature.

[65] Zhao K., Wang W., Rando O.J., Xue Y., Swiderek K., Kuo A., Crabtree G.R. (1998). Rapid and phosphoinositol-dependent binding of the SWI/SNF-like BAF complex to chromatin after T lymphocyte receptor signaling. Cell.

[66] Ramirez-Carrozzi V.R., Nazarian A.A., Li C.C., Gore S.L., Sridharan R., Imbalzano A.N., Smale S.T. (2006). Selective and antagonistic functions of SWI/SNF and MI-2beta nucleosome remodeling complexes during an inflammatory response. Genes Dev..

[67] Ramirez-Carrozzi V.R., Braas D., Bhatt D.M., Cheng C.S., Hong C., Doty K.R., Black J.C., Hoffmann A., Carey M., Smale S.T. (2009). A unifying model for the selective regulation of inducible transcription by CPG islands and nucleosome remodeling. Cell.

[68] Lomvardas S., Thanos D. (2002). Modifying gene expression programs by altering core promoter chromatin architecture. Cell.

[69] Rogatsky I., Adelman K. (2014). Preparing the first responders: Building the inflammatory transcriptome from the ground up. Mol. Cell.

[70] Yamaguchi Y., Shibata H., Handa H. (2013). Transcription elongation factors DSIF and NELF: Promoter-proximal pausing and beyond. Biochim. Biophys. Acta.

[71] Barboric M., Nissen R.M., Kanazawa S., Jabrane-Ferrat N., Peterlin B.M. (2001). NF-kappaB binds p-tefb to stimulate transcriptional elongation by RNA polymerase II. Mol. Cell.

[72] Takahashi H., Parmely T.J., Sato S., Tomomori-Sato C., Banks C.A., Kong S.E., Szutorisz H., Swanson S.K., Martin-Brown S., Washburn M.P. (2011). Human mediator subunit med26 functions as a docking site for transcription elongation factors. Cell.

[73] Prinjha R., Tarakhovsky A. (2013). Chromatin targeting drugs in cancer and immunity. Genes Dev..

[74] Calo E., Wysocka J. (2013). Modification of enhancer chromatin: What, how, and why?. Mol. Cell.

[75] Spitz F., Furlong E.E. (2012). Transcription factors: From enhancer binding to developmental control. Nat. Rev. Genet..

[76] Agarwal S., Avni O., Rao A. (2000). Cell-type-restricted binding of the transcription factor NFAT to a distal IL-4 enhancer *in vivo*. Immunity.

[77] Zhou L., Nazarian A.A., Xu J., Tantin D., Corcoran L.M., Smale S.T. (2007). An inducible enhancer required for IL12b promoter activity in an insulated chromatin environment. Mol. Cell. Biol..

[78] De Santa F., Barozzi I., Mietton F., Ghisletti S., Polletti S., Tusi B.K., Muller H., Ragoussis J., Wei C.L., Natoli G. (2010). A large fraction of extragenic RNA pol II transcription sites overlap enhancers. PLoS Biol..

[79] Lam M.T., Cho H., Lesch H.P., Gosselin D., Heinz S., Tanaka-Oishi Y., Benner C., Kaikkonen M.U., Kim A.S., Kosaka M. (2013). Rev-erbs repress macrophage gene expression by inhibiting enhancer-directed transcription. Nature.

[80] Melo C.A., Drost J., Wijchers P.J., van de Werken H., de Wit E., Oude Vrielink J.A., Elkon R., Melo S.A., Leveille N., Kalluri R. (2013). Ernas are required for p53-dependent enhancer activity and gene transcription. Mol. Cell.

[81] Lai D., Wan M., Wu J., Preston-Hurlburt P., Kushwaha R., Grundstrom T., Imbalzano A.N., Chi T. (2009). Induction of TLR4-target genes entails calcium/calmodulin-dependent regulation of chromatin remodeling. Proc. Natl. Acad. Sci. USA.

[82] Bhatt D.M., Pandya-Jones A., Tong A.J., Barozzi I., Lissner M.M., Natoli G., Black D.L., Smale S.T. (2012). Transcript dynamics of proinflammatory genes revealed by sequence analysis of subcellular RNA fractions. Cell.

[83] Danko C.G., Hah N., Luo X., Martins A.L., Core L., Lis J.T., Siepel A., Kraus W.L. (2013). Signaling pathways differentially affect RNA polymerase ii initiation, pausing, and elongation rate in cells. Mol. Cell.

[84] Barnes P.J. (2009). Targeting the epigenome in the treatment of asthma and chronic obstructive pulmonary disease. Proc. Am. Thorac. Soc..

[85] Bayarsaihan D. (2011). Epigenetic mechanisms in inflammation. J. Dent. Res..

[86] Saccani S., Natoli G. (2002). Dynamic changes in histone H3 LYS 9 methylation occurring at tightly regulated inducible inflammatory genes. Genes Dev..

[87] De Santa F., Totaro M.G., Prosperini E., Notarbartolo S., Testa G., Natoli G. (2007). The histone H3 lysine-27 demethylase Jmjd3 links inflammation to inhibition of polycomb-mediated gene silencing. Cell.

[88] De Santa F., Narang V., Yap Z.H., Tusi B.K., Burgold T., Austenaa L., Bucci G., Caganova M., Notarbartolo S., Casola S. (2009). Jmjd3 contributes to the control of gene expression in lps-activated macrophages. EMBO J..

[89] Barski A., Cuddapah S., Cui K., Roh T.Y., Schones D.E., Wang Z., Wei G., Chepelev I., Zhao K. (2007). High-resolution profiling of histone methylations in the human genome. Cell.

[90] Jones P.A., Liang G. (2009). Rethinking how DNA methylation patterns are maintained. Nat. Rev. Genet..

[91] Cheung H.H., Lee T.L., Rennert O.M., Chan W.Y. (2009). DNA methylation of cancer genome. Birth Defects Res. C Embryo Today.

[92] Shuto T., Furuta T., Oba M., Xu H., Li J.D., Cheung J., Gruenert D.C., Uehara A., Suico M.A., Okiyoneda T. (2006). Promoter hypomethylation of toll-like receptor-2 gene is associated with increased proinflammatory response toward bacterial peptidoglycan in cystic fibrosis bronchial epithelial cells. FASEB J..

[93] Heintzman N.D., Stuart R.K., Hon G., Fu Y., Ching C.W., Hawkins R.D., Barrera L.O., van Calcar S., Qu C., Ching K.A. (2007). Distinct and predictive chromatin signatures of transcriptional promoters and enhancers in the human genome. Nat. Genet..

[94] Visel A., Blow M.J., Li Z., Zhang T., Akiyama J.A., Holt A., Plajzer-Frick I., Shoukry M., Wright C., Chen F. (2009). Chip-seq accurately predicts tissue-specific activity of enhancers. Nature.

[95] Lefevre P., Witham J., Lacroix C.E., Cockerill P.N., Bonifer C. (2008). The LPS-induced transcriptional upregulation of the chicken lysozyme locus involves CTCF eviction and noncoding RNA transcription. Mol. Cell.

[96] Zaret K.S., Watts J., Xu J., Wandzioch E., Smale S.T., Sekiya T. (2008). Pioneer factors, genetic competence, and inductive signaling: Programming liver and pancreas progenitors from the endoderm. Cold Spring Harb. Symp. Quant. Biol..

[97] Xu J., Watts J.A., Pope S.D., Gadue P., Kamps M., Plath K., Zaret K.S., Smale S.T. (2009). Transcriptional competence and the active marking of tissue-specific enhancers by defined transcription factors in embryonic and induced pluripotent stem cells. Genes Dev..

[98] Foster S.L., Hargreaves D.C., Medzhitov R. (2007). Gene-specific control of inflammation by TLR-induced chromatin modifications. Nature.

[99] Lane D., Levine A. (2010). P53 research: The past thirty years and the next thirty years. Cold Spring Harb. Perspect. Biol..

[100] Menendez D., Shatz M., Azzam K., Garantziotis S., Fessler M.B., Resnick M.A. (2011). The toll-like receptor gene family is integrated into human DNA damage and p53 networks. PLoS Genet..

[101] Lowe J.M., Menendez D., Bushel P.R., Shatz M., Kirk E.L., Troester M.A., Garantziotis S., Fessler M.B., Resnick M.A. (2014). P53 and NF-kappaB coregulate proinflammatory gene responses in human macrophages. Cancer Res..

[102] Tan C., Mui A., Dedhar S. (2002). Integrin-linked kinase regulates inducible nitric oxide synthase and cyclooxygenase-2 expression in an NF-kappa B-dependent manner. J. Biol. Chem..

[103] Makino K., Day C.P., Wang S.C., Li Y.M., Hung M.C. (2004). Upregulation of IKKalpha/IKKbeta by integrin-linked kinase is required for Her2/Neu-induced NF-kappaB antiapoptotic pathway. Oncogene.

[104] Agouni A., Sourbier C., Danilin S., Rothhut S., Lindner V., Jacqmin D., Helwig J.J., Lang H., Massfelder T. (2007). Parathyroid hormone-related protein induces cell survival in human renal cell carcinoma through the pi3k AKT pathway: Evidence for a critical role for integrin-linked kinase and nuclear factor kappa B. Carcinogenesis.

[105] Medici D., Nawshad A. (2010). Type I collagen promotes epithelial-mesenchymal transition through ILK-dependent activation of NF-kappab and LEF-1. Matrix Biol..

[106] Del Nogal M., Luengo A., Olmos G., Lasa M., Rodriguez-Puyol D., Rodriguez-Puyol M., Calleros L. (2012). Balance between apoptosis or survival induced by changes in extracellular-matrix composition in human mesangial cells: A key role for ILK-NFkappab pathway. Apoptosis.

[107] Wani A.A., Jafarnejad S.M., Zhou J., Li G. (2011). Integrin-linked kinase regulates melanoma angiogenesis by activating NF-kappaB/interleukin-6 signaling pathway. Oncogene.

[108] Han C., Jin J., Xu S., Liu H., Li N., Cao X. (2010). Integrin CD11b negatively regulates TLR-triggered inflammatory responses by activating Syk and promoting degradation of MyD88 and TRIF via Cbl-b. Nat. Immunol..

[109] Wang L., Gordon R.A., Huynh L., Su X., Park Min K.H., Han J., Arthur J.S., Kalliolias G.D., Ivashkiv L.B. (2010). Indirect inhibition of toll-like receptor and type I interferon responses by itam-coupled receptors and integrins. Immunity.

[110] Yee N.K., Hamerman J.A. (2013). Beta2 integrins inhibit TLR responses by regulating NF-kappaB pathway and p38 MAPK activation. Eur. J. Immunol..

[111] Kagan J.C., Medzhitov R. (2006). Phosphoinositide-mediated adaptor recruitment controls toll-like receptor signaling. Cell.

[112] Medvedev A.E., Flo T., Ingalls R.R., Golenbock D.T., Teti G., Vogel S.N., Espevik T. (1998). Involvement of CD14 and complement receptors CR3 and CR4 in nuclear factor-kappaB activation and TNF production induced by lipopolysaccharide and group B streptococcal cell walls. J. Immunol..

[113] Perera P.Y., Mayadas T.N., Takeuchi O., Akira S., Zaks-Zilberman M., Goyert S.M., Vogel S.N. (2001). CD11b/CD18 acts in concert with CD14 and toll-like receptor (TLR) 4 to elicit full lipopolysaccharide and taxol-inducible gene expression. J. Immunol..

[114] Petzold T., Orr A.W., Hahn C., Jhaveri K.A., Parsons J.T., Schwartz M.A. (2009). Focal adhesion kinase modulates activation of NF-kappaB by flow in endothelial cells. Am. J. Physiol. Cell Physiol..

[115] Kwok T., Zabler D., Urman S., Rohde M., Hartig R., Wessler S., Misselwitz R., Berger J., Sewald N., Konig W. (2007). Helicobacter exploits integrin for type IV secretion and kinase activation. Nature.

[116] Tang Q., Zhao S., Wu J., Zheng F., Yang L., Hu J., Hann S.S. (2015). Inhibition of integrin-linked kinase expression by emodin through crosstalk of ampkalpha and Erk1/2 signaling and reciprocal interplay of sp1 and C-jun. Cell. Signal..

[117] Wang W., Liu Q., Zhang Y., Zhao L. (2014). Involvement of Ilk/erk1/2 and Ilk/p38 pathways in mediating the enhanced osteoblast differentiation by micro/nanotopography. Acta Biomater..

[118] Yu L., Yuan X., Wang D., Barakat B., Williams E.D., Hannigan G.E. (2014). Selective regulation of p38beta protein and signaling by integrin-linked kinase mediates bladder cancer cell migration. Oncogene.

[119] Hashiramoto A., Murata M., Kawazoe T., Yoshida K., Akiyama C., Shiozawa K., Shiozawa S. (2011). Heat shock protein 90 maintains the tumour-like character of rheumatoid synovial cells by stabilizing integrin-linked kinase, extracellular signal-regulated kinase and protein kinase B. Rheumatology.

[120] Noh K.T., Park Y.M., Cho S.G., Choi E.J. (2011). Gsk-3beta-induced ask1 stabilization is crucial in Lps-induced endotoxin shock. Exp. Cell Res..

